# Preparation and Component Optimization of Resin-Based Permeable Brick

**DOI:** 10.3390/ma13122701

**Published:** 2020-06-13

**Authors:** Xiaofu Wang, Xiong Zhang

**Affiliations:** Key Laboratory of Advanced Civil Engineering Materials of Education Ministry, School of Material Science and Technology, Tongji University, 4800 Cao’an Road, Shanghai 201804, China; zhangxiong@tongji.edu.cn

**Keywords:** permeable brick, epoxy resin, desert sand, particle size distribution, SEM

## Abstract

The present study aims to prepare resin-based permeable bricks with micron-sized pores using fine aggregate with a particle diameter of 0.08–0.6 mm and bisphenol-A epoxy resin, a polymer binder. The properties of the binder, the characteristic parameters of the aggregate, and the micro pore structure of the brick were studied in order to break through the limitations of traditional porous permeable materials. The dynamic mechanical properties of resin were analyzed by dynamic mechanical analysis (DMA). The frequency parameter of particle size of 10 kinds of aggregate from different regions were obtained by digital image processing, and the characteristic parameter (aggregate distribution coefficient α) was obtained by modified Gaussian distribution. The microstructure of porous brick was analyzed by scanning electron microscopy-energy-dispersive X-ray spectroscopy (SEM-EDS). The test results show: (1) the glass transition temperature (Tg) of the resin is 61 °C; (2) the parameters of aggregate particle group will affect the performance of porous permeable materials; (3) the minimum effective pore diameter of the permeable brick is 30 μm, the maximum permeable rate is $6.22\times 10^{-2}\;\mathrm{cm}/s$ and the compressive strength is 41.08 MPa. The conclusions of this study will provide an important reference for permeable materials in the micron-scale pore range and the selection of binder and aggregate materials.

## 1. Introduction

As a result of the process of global urbanization, more than 50% of the world’s population now live in cities [1]. Cities are often built near water sources such as rivers, which bring drinking water and transportation to people. However, with the expansion of the city, the total impervious area of natural water sources gradually increases in the form of hardened ground, roof, lawn, etc. This will lead to the reduction of water source filterability and the change of sediment and solute export in the global river basin, which will seriously affect the natural environment [2]. Furthermore, many urban diseases have emerged during urban expansion, for example: air pollution caused by the concentration of transportation and industry; eutrophication and change of hydrological system caused by point source pollution into waters; changes in urban biodiversity [3]. Among such urban diseases, the typical ones are “urban pluvial flooding” caused by urban hydrological change and “heat island effect” caused by urban atmospheric change.

“Urban pluvial flooding” refers to the phenomenon of waterlogging disaster in the city caused by heavy precipitation or continuous precipitation exceeding the urban drainage capacity. There are pluvial flooding and surface runoffs in many cities around the world [4,5,6,7], and this reflects the hydrological and environmental impact of urban expansion that increase the runoff rate and flow, reduce the soil water that usually supports infiltration, reduce groundwater supply and base flow, and reduce evapotranspiration [8].

The heat island effect refers to the phenomenon of urban microclimate change with the increase of urban heat waves. This phenomenon is due to man-made reasons, which change the local temperature, humidity, air convection and other factors of the urban surface and buildings. It results in reduced sensible heat convection and urban land evaporative cooling during the daytime, the use of energy and the heat released by solar energy stored in buildings make the city warm during the night [9,10,11,12].

Urban diseases affect the natural ecological environment, they also threaten the safety of people living in cities, and they cause immeasurable economic losses. It is urgent to solve these problems. Since the 1970s, lots of developed countries (e.g., USA, Germany, United Kingdom, Japan, Singapore and Australia) have put forward targeted programs to guide urban planning and construction, e.g., best management practices (BMP), low impact development (LID), green infrastructure (GI), sustainable urban drainage system (SUDS), and water sensitive urban design (WSUD), low impact urban design and development (LIUDD), active beautiful and clean waters program (ABC) [13,14,15,16,17,18,19], and these plans combine municipal engineering, urban hydrology, environmental science, social science, etc., and are undertaken at the planning level to the specific construction level. In previous studies [20,21], it can be seen that in the process of urbanization, the hardened road surface gradually replaced the ecological road surface, which aggravated the urban pluvial flooding and heat island effects, and the impervious surface replaced the previous surface. An ecological pavement is capable of absorbing, accumulating and slow-releasing natural water, it can supplement soil water and groundwater, enhance convection and evaporation, and reduce urban heat waves. By contrast, the surface-hardened pavement is an aggregate of asphalt pavement, cement concrete pavement, etc. It usually does not have the functions of ecological pavement, so they have a negative correlation with the treatment of urban diseases. Urbanization is an irreversible process for a region. To solve the aforementioned urban diseases, urban roads must have both mechanical and ecological functions. According to this idea, researchers [22] proposed a porous permeable pavement system (PPS), which typically includes porous concrete, permeable asphalt, clay permeable brick and cement-based permeable brick [23,24,25,26,27,28], and their corresponding scenarios are light load urban roadway and pedestrian footpath that can not only bear the load of an urban pavement, but also have the function of water permeability, drainage and water storage, which can solve the contradiction between hardened pavement and ecological pavement. This kind of material also plays a role of filtration in promoting hydrological recovery in urban environment. But it also has corresponding disadvantages, e.g., it has a large surface-exposed pores, it is easy to block, has a short life cycle, has a high cost in terms of use and maintenance, and has an unsightly appearance, etc. [29], and these defects hinder the promotion and use of porous permeable materials.

The above phenomenon is often encountered in the use of PPS, and the problems behind it are that the composition of such materials, aggregates and binder materials, are limited to common materials, and performance design and material selection cannot be based on the required pore diameter, water permeability, strength, etc. Research has been undertaken to explore the relationship between pore size and permeability [30], Because the Reynolds number of liquid flowing in this kind of porous media is generally less than 10, all of them are in laminar flow state, so the permeability of permeable materials can be calculated by using the Kozeny–Carman equation. In all of the permeable materials with laminar flow state, it is satisfied that with the increase of pore diameter, the effective porosity will increase, and the permeability will also be improved and, therefore, permeable concrete and permeable asphalt with large pore size have higher water permeability. However, the pore size of the exposed surface is too large, which leads to pore blocking, stone falling and other problems. The permeable pavement material with a pore size of millimeters or even smaller can overcome the aforementioned defects, and has a good consideration of water permeability, water storage, bearing, long-term performance, stability and other properties.

In order to reduce the pore size, fine aggregates can be used to prepare water-permeable materials. The fine aggregate permeable brick mainly includes cement-based permeable brick and sintered permeable brick [31,32,33], researchers have prepared small pore size cement-based permeable bricks using aggregates with a particle size of 3–5 mm [24], and sintered permeable bricks with small pore diameters were prepared from fine aggregates with a particle size of 0.85–2 mm [31]. The sintering process of clay permeable brick has high energy consumption and is not environmentally friendly, there will also be secondary pollution even if waste slag and waste glass are used to make bricks. At the same time, quartz, which is the main component of sintered permeable bricks, will expand in volume during the sintering process, so a large number of microcracks will be produced, which exist in the crystal interior and the contact part between particles and glass phase. The existence of microcracks reduces the strength of the crystal under stress, while the brick has a large volume, and the accumulated deformation and cracks will also reduce the strength and service life of the brick. For cement-based permeable brick, it is difficult to balance the strength and content of cementitious materials, at the same time, the cement-based permeable brick has a long curing time due to the limitation of the cementitious material, which is not conducive to turnover and storage in industrialization. Therefore, materials that are environmentally friendly and have balanced strength and dosage need to be studied urgently.

To regulate the properties of permeable bricks, the constituent materials such as binder and aggregate must be studied. Digital image processing (DIP) is more convenient and accurate to obtain the particle group parameters of aggregate when the fine aggregate is used to prepare permeable brick. The particle group parameters of aggregate are the key to coupling the permeable rate and mechanical properties of permeable brick, such as the median diameter, distribution, roundness, etc. [34,35], but there is no such research on permeable bricks with micron-sized pores. In the use of binder, the conventional thinking is to use inorganic cementitious materials such as cement. Undoubtedly, this kind of material is widely used and mature in large-pore permeable materials. However, in order to meet the performance requirements of small-pore permeable bricks, a new binder is required, which should have the properties of short curing time and small amount of admixture (small volume proportion) but can provide enough strength and strong pressure-bearing capacity. In fact, it is not difficult to think of resin binders with excellent bonding and pressure-bearing capabilities in commonly used binder materials.

The innovation of this study is as follows:

A kind of permeable brick was developed, which uses organic material as binder and fine sand with particle size in the range of 0.08–0.6 mm as aggregate. It can be compatible with the performance of hardened pavements and ecological pavements. Its performance in all aspects exceeds the national standard GB/T 25993-2010 [36], and the parameters stipulated in national standard are shown in Table 1. In order to distinguish it from cement-based permeable brick and sintered permeable brick, this kind of brick is named resin-based permeable brick (RBPB).

In this study, the performance of resin binder material was tested, and the particle group parameters of aggregate were obtained. On this basis, the parameters and the related performance of permeable materials were analyzed, and the preparation and aggregate optimization method of micron porous permeable brick were obtained. Scanning electron microscopy (SEM) was used to observe the micro pore characteristics, binder material characteristics and aggregate defects. It has reference value for the research and design of permeable pavement materials.

## 2. Experimental

Resin-based permeable brick is a kind of pavement material with water permeability function made of fine sand as aggregate and resin as binder after non-sintered molding process. RBPB has a simple forming process and can be carried out at normal temperature. It has a short curing time, micropores, compact structure and good appearance after molding.

### 2.1. Raw Material

Binders and aggregates are materials for making resin-based permeable pavement tiles. Binders: AB-type epoxy resin (two components), component A is bisphenol-A epoxy resin, a white transparent viscous liquid, with epoxy value of 0.44, and the industrial brand is E44; component B is amine curing agent (adduct of diethylenetriamine and butyl glycidyl ether), whose industrial grade is 593, relative density is 0.985, viscosity (25 °C) is 90~150 mPa·s, with a total amine value is 500~700 mgKOH/g, which is a yellow transparent liquid. The reason for choosing these two components is that the mechanical properties and high temperature resistance of E44 epoxy resin are better than those of other bisphenol-A epoxy resin and Polyurethane (PU) resin. Among many curing agents, only the alkaline curing agent containing aliphatic diamine and E44 can be used together to optimize the mechanical properties of RBPB. Because the ultimate goal of RBPB is industrial production and application, considering the cost and mechanical properties, E44 was selected as the component A, and the alkaline curing agent 593 was used as the component B.

The curing of the epoxy resin needs to be performed under dry conditions. In a dry environment at room temperature, the 1-day strength can reach 90% of the maximum value. According to the source of production, the sand for construction can be divided into artificial sand, natural sand and mixed sand. In this paper, according to the idea of selecting representative natural sand, 10 kinds of different aggregates with similar particle size but different distribution were selected as the analysis object. Both artificial sand and mixed sand are not in the scope of this paper. The natural sand can be divided into desalinated sea sand, mountain sand, Lake sand, river sand and aeolian sand. 10 kinds of sand samples are selected as the aggregate for RBPB preparation in the experiment. The sand source of the above natural sand comes from the main sand-producing areas in China, such as Xiamen, Sanming, Liaoning, Inner Mongolia, Chengdu, Chongqing, Shanghai, Jiangyan, etc. Although the mineral composition of these sand samples is different, the main purpose of using these aggregates is to use them as aggregates of permeable materials to build pores and mechanical load. According to the methods in GB/T 14684-2011 “Sand for Construction” [37], it can be ascertained that the above sand sample’s firmness index and crushing index are all grade I. In the immersion test of sodium sulfate solution, their mass loss ≤8%. The maximum single-stage crushing index ≤20%. Their particle size is controlled within 0.08–0.6 mm by vibrating screen.

### 2.2. Sample Preparation

**Figure 1:**
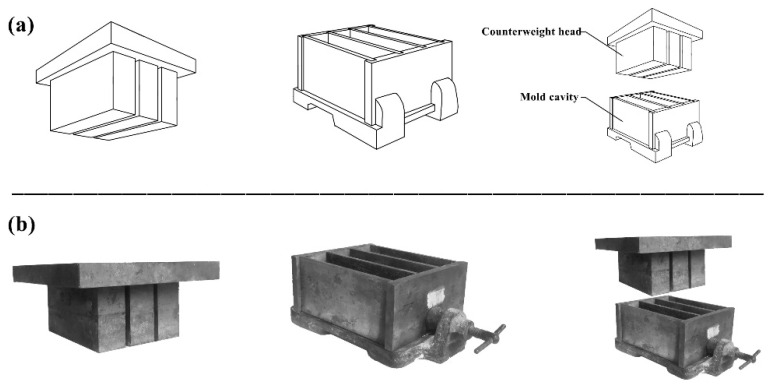
Special mold. (**a**) Design drawing. (**b**) Real mold.

For the preparation of RBPB, firstly, a certain amount of sand shall be weighed according to the formula and poured into the mixer, epoxy resin and curing agent shall be added, and then the mixture shall be placed in the mixer for 240 s until the materials are well mixed. Since the mixture does not have a self-leveling property, it needs to be compacted. In order to make the pores well-distributed, a vibration compaction method is adopted. In this study, a special cavity and a 60 kg counterweight head are designed, as shown in Figure 1. We pour the mixture into the mold cavity, fully insert and tamp the mixture with a scraper, cover the mold cavity with a counterweight head and place it on a concrete vibration table with a vibration frequency of 50 (±3) Hz, and vibrate it for 90 s to press the mixture into shape. After 24 h of natural curing, the mold can be removed, and the size of the permeable brick sample is 300 mm × 100 mm × 50 mm.

The proportion is shown in Table 2.

When the total volume of porous materials is constant, the mechanical properties increase with the increase of the content of binder materials, but the water permeability will decrease. Through some exploration experiments, it was found that the mechanical properties cannot meet the requirements when the mass ratio of the binder is lower than 2%, and the water permeability cannot meet the requirements when the mass ratio is higher than 8%. Therefore, considering the mechanical properties, water permeability and cost, the proportion of the total mass of resin and curing agent accounting for 6% of the aggregate is used for subsequent research, and the proportion of curing agent and resin is as follows:

(1) m(curing agent):m(resin)=3:10

### 2.3. Test Methods

#### 2.3.1. Test Method for Water Permeability and Mechanical Properties of Resin-Based Permeable Brick (RBPB)

As there is no standard for “resin-based” water-permeable materials, the test methods and indicators of water-permeable properties of permeable materials are based on national standard GB/T 25993-2010 [38] for ordinary permeable brick. The sample was drilled into three φ 75 mm × 50 mm cylinders and tested with a constant water head tester (Senzhong Experimental Instrument Co., Ltd., Cangzhou, China) that conforms to Darcy’s law, and the permeability coefficient K($\mathrm{cm}\cdot s^{-1}$) is calculated by the formula as shown in Equation (2). The compressive strength of the samples was measured by using a universal testing machine at a crosshead speed of 2 mm/min [31]. Each group has three bricks, each brick is drilled into three samples, and the test value is taken as the average. The flexural strength was measured according to the loading rate of 0.04~0.06 MPa/s [39].

(2) $$K=\frac{QL}{AHt}$$

where: Q(mL) is the volume of effluent water in time, t. L (cm) is the thickness of the sample, A (cm^2^) is the upper surface area of the sample, H (cm) is the difference of water level, and t (s) is time.

#### 2.3.2. Tensile Test

Binder materials determine the mechanical properties and durability of permeable materials. In order to accurately study the intrinsic properties of resin materials, it is necessary to study their macroscopic and thermodynamic properties respectively. Therefore, the methods of tensile test and dynamic mechanical analysis (DMA) are selected to carry out experimental research on resin materials, which will help to provide guidance for the use and design of resin materials.

The preparation method of resin casting test piece refers to the national standard GB/T 2567-2008 [40]; we first prepared the mold according to the standard sample size, then configured and pour the test materials according to the proportion in Table 3 at the temperature of 15~30 °C and the relative humidity of less than 75%, placed them at room temperature for 48 h, placed them at room temperature for 504 h (including sample processing time) after demoulding, then polished the standard size of the sample, and finally performed the tensile test.

**Figure 2:**
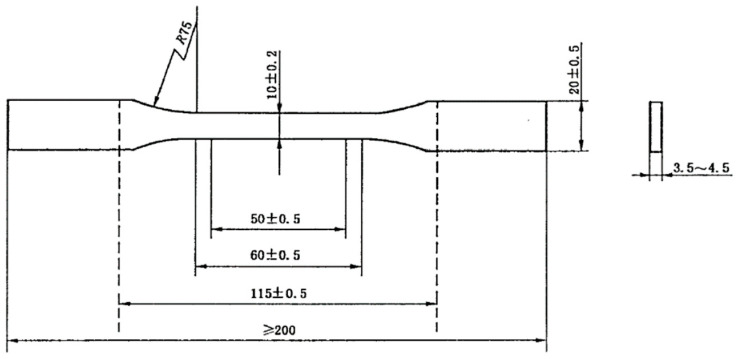
Tensile sample (unit: mm).

The tensile strength test speed was 10 mm/min, and the arbitration test speed was 2 mm/min. The test measured five samples at a time, and the experimental results were averaged. The size of the tensile sample is shown in Figure 2.

**Figure 3:**
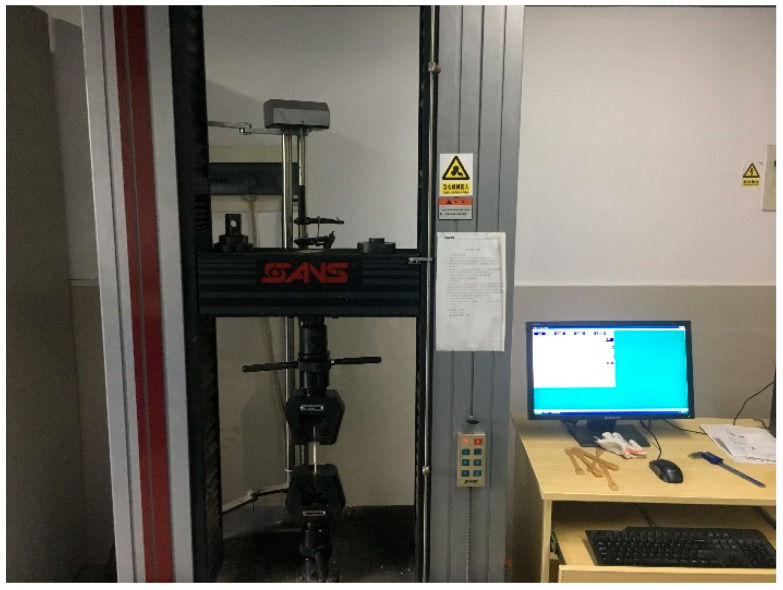
Universal testing machine.

The tensile test of the resin castings used the SANS CMT7000 universal testing machine (MTS, Eden Prairie, MN, USA), as shown in Figure 3.

#### 2.3.3. Dynamic Mechanical Analysis (DMA)

DMA tests were conducted on a TA Q800 dynamic mechanical analyser (TA Instruments, New Castle, DE, USA) at the frequency of 1 Hz, temperature range of 20–150 °C, and the heating rate was set at 2 Kmin^−1^, and the modulus test was three-point bending. The size of the specimens was 5 mm × 10 mm × 50 mm. The viscosity-temperature behavior was observed and the glass transition temperature (Tg) points were calculated.

#### 2.3.4. Particle Group Parameter Test

**Figure 4:**
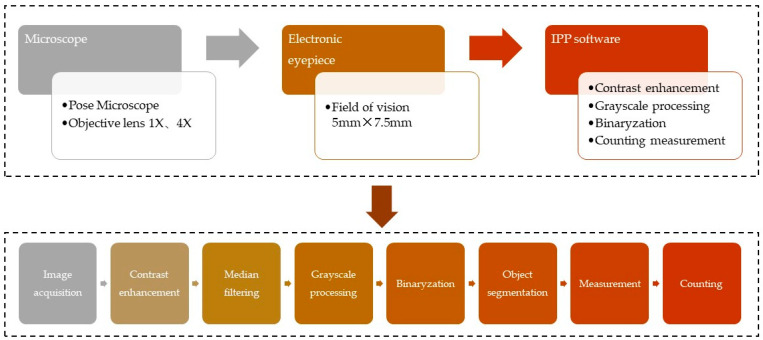
Image acquisition and analysis process.

The optical stereo microscope and Sony ILCE-6000 24.3 megapixel camera (SONY, Tokyo, Japan) were used to collect the image information of the sand particle group, and then the image information of the particle group was analyzed and processed with Image Pro Plus (IPP) software to obtain the particle group parameters. Figure 4 shows the image collection and analysis process.

**Figure 5:**
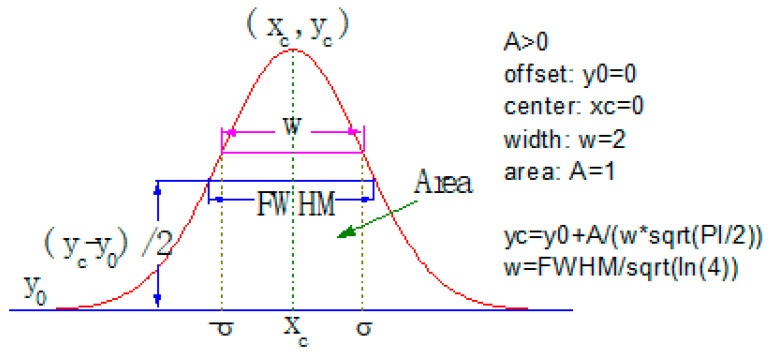
Modified Gaussian distribution curve.

A modified Gaussian distribution function is used to describe the particle size distribution of the aggregates. At the same time, data such as the most probable particle size and the standard deviation of the distribution can be obtained. Based on these data, the aggregate samples can be targeted better. The modified Gaussian distribution curve is shown in Figure 5 and the equation is shown in Equation (3) below. It can be seen that in the ideal state, it is still a standard normal distribution.

#### 2.3.5. Scanning Electron Microscopy (SEM) Analysis

**Figure 6:**
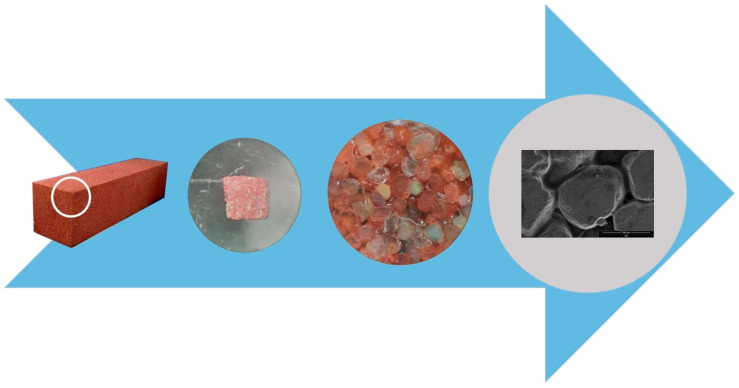
Sample preparation, grinding and image acquisition process.

Take samples from a bigger brick strip, cut and polish them to a cube with the size of 5 mm × 5 mm × 2.5 mm, then use the ultrasonic cleaner to clean them for 60 s to complete the dust removal. The permeable brick was cut into 5 mm × 5 mm × 2.5 mm samples, and use scanning electron microscope ‘Nova Nano SEM 450’ (FEI, Hillsboro, OR, USA) to observe the samples. The research method is shown in Figure 6. In order to facilitate the microscopic observation of sand particle shape, a small amount of red pigment was added to the brick-making process.

## 3. Results and Discussion

### 3.1. Test Results of Binder Materials

#### 3.1.1. Tensile Test

**Figure 7:**
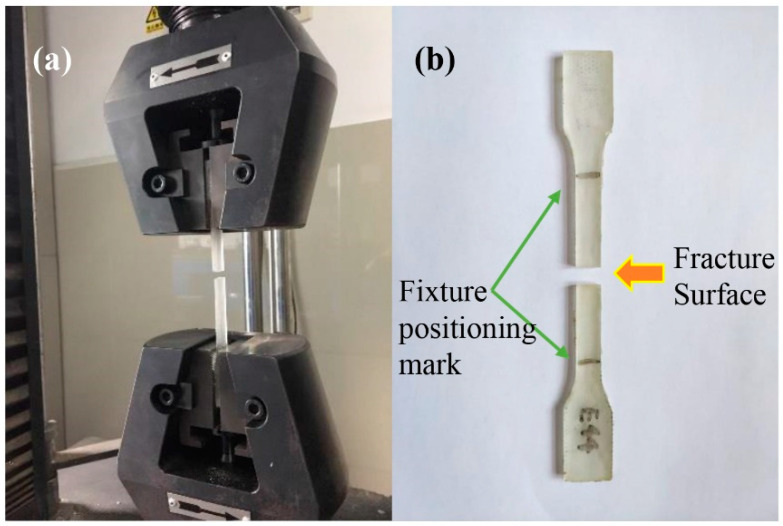
Tensile test results. (**a**) Test process. (**b**) Fractured specimen in the test.

**Figure 8:**
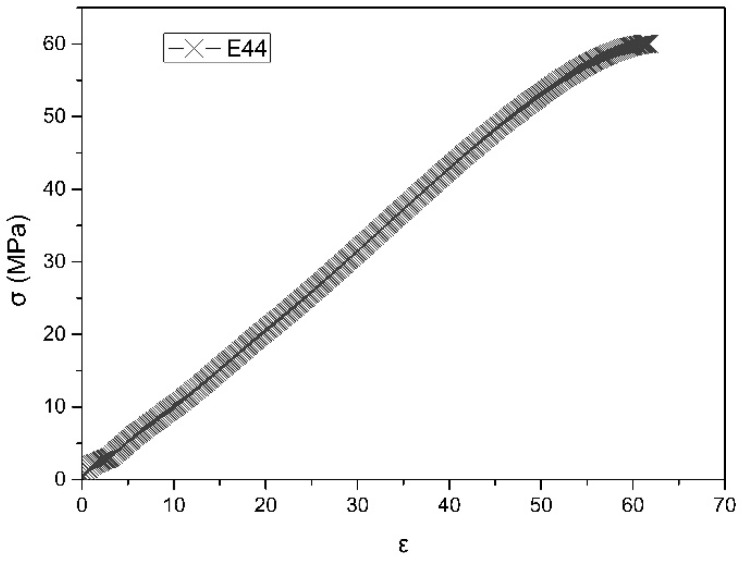
Stress-strain curve of resin specimen.

From the pulled resin specimen showed in Figure 7a,b, it can be seen that there is no obvious elongation and necking at the fracture. The stress-strain curve of the resin is shown in Figure 8. From the curve, it can be seen that this epoxy resin only has the elastic deformation stage but not the yield stage during the tensile fracture, so the selected epoxy resin is a brittle material. In building materials, brittle materials are often used for bearing pressure, such as concrete [41], so this property of the resin selected in this paper can meet the demand of a pressure-bearing pavement brick. The data of tensile test is shown in Table 4, the elongation at break is only 6.17%, and the tensile stress at break is 60.03 MPa, which proves that it has excellent mechanical properties as the binder of permeable brick.

#### 3.1.2. DMA

**Figure 9:**
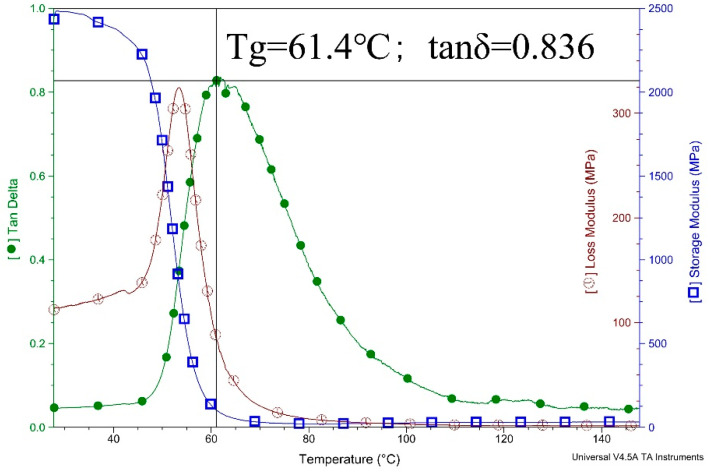
Dynamic thermomechanical analysis of binder material E44.

Dynamic mechanical indexes of bisphenol-A epoxy resin can be obtained by dynamic mechanical analysis, such as storage modulus (E′), which indicates the elasticity of epoxy resin, and loss modulus (E″), which indicates the plasticity of epoxy resin, glass transition temperature (Tg) and loss factor (tanδ), as shown in Figure 9.

Figure 9 also shows that the glass transition temperature of bisphenol-A epoxy resin is 61.4 °C, and tanδ = 0.836. Temperature change will severely affect the hydrogen bond and Van der Waals force will also lead to the change of chain segment mobility. Therefore, under the glass transition temperature, the polymer is in the glass state, and the molecular chain and chain segment cannot move, but the atoms (or groups) that make up the molecule vibrate at their equilibrium position; while at the glass transition temperature, the molecular chain cannot move, but the chain segment begins to move, showing a high elastic property. If the temperature rises again, the whole molecular chain will move and show a viscous flow property [42]. Tg is the boundary between the glassy state and the high elasticity state of the polymer. When the temperature exceeds Tg, the polymer will undergo a large deformation once it is stressed, which will seriously affect its strength. Therefore, the higher the glass transition temperature, the more stable the mechanical properties of the resin can be during use. Therefore, it can be judged that the applicable temperature of the cured resin is less than 61 °C, and the binder material of RBPB can meet the requirements of the application scenario. Through the tanδ value, we can learn that the molecular chain of the resin is rigid.

### 3.2. Particle Group Parameter Test Results of Aggregate

#### 3.2.1. Frequency Analysis of Particle Size

**Figure 10:**
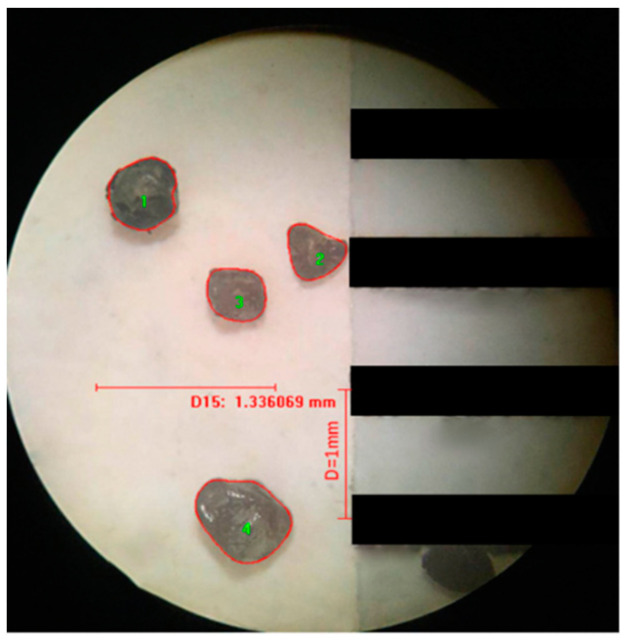
Conversion processing of particle size.

**Figure 11:**
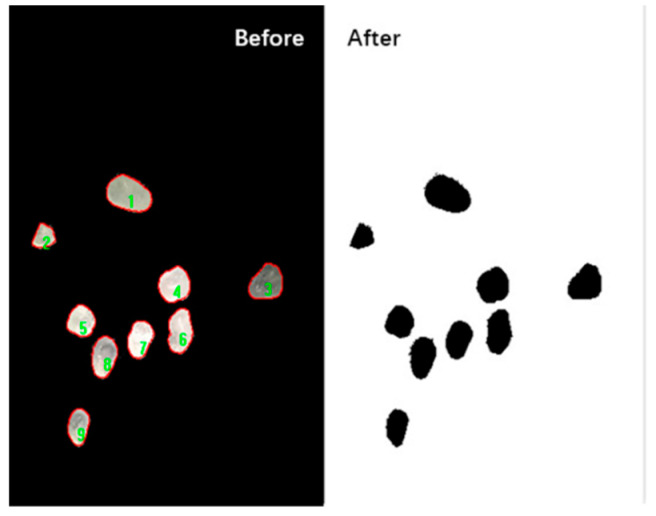
Image binarization.

Since the unit of the numerical value output by the image processing software is a pixel, the actual size and the pixel value size need to be converted, and the standard of unit conversion is shown in Figure 10. The acquired particle image is binarized by Image Pro Plus software to extract sand data. Figure 11 is the schematic diagram of sand image extracted by microscope and binarization conversion.

**Figure 12:**
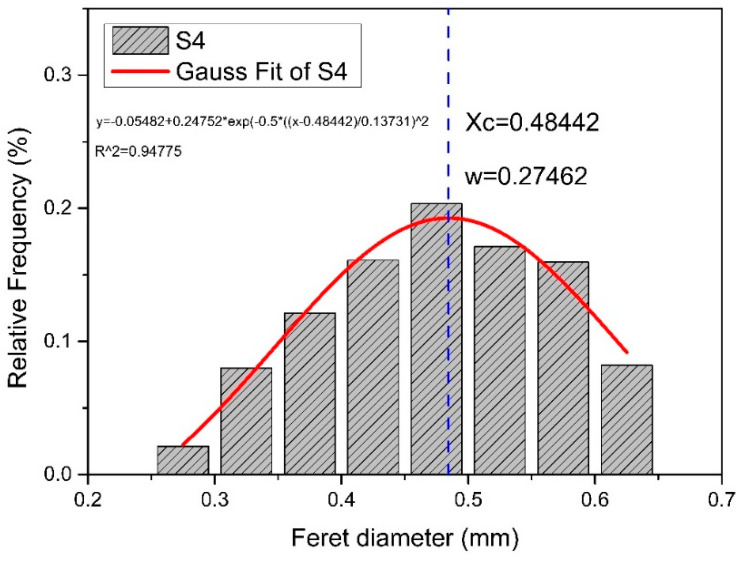
Gauss equation fitting curve of particle size distribution.

The particle size distribution of particle groups is discontinuous, but the particle size distribution of a large number of particle groups can be approximately considered as continuous. Therefore, according to the IPP image analysis and processing, the frequency distribution of sand sample size can be obtained. Through the modified Gaussian distribution function, the curve can be fitted on the basis of frequency distribution, and the most probable particle size $x_{c}$ and distribution standard deviation w can be obtained as shown in Figure 12.

The modified normal distribution density function is as Equation (3):

(3) $$y\;=y_{0}+\frac{A}{w\sqrt{\pi /2}}e^{-2\frac{{\left(x-x_{c}\right)}^{2}}{w^{2}}}$$

where: $x_{c}$ (mm) is the most probable particle size of the aggregate and w is the distribution standard deviation of the aggregate.

The distribution standard deviation w in the formula reflects the dispersion degree of particle size to $x_{c}$. Define α as the aggregate distribution coefficient, which is a dimensionless quantity, and the calculation formula of α is shown in Equation (4).

(4) $$\alpha \;=\frac{w}{x_{c}}$$

Two parameters $x_{c}$ and w in the modified Gaussian distribution function determine the particle size distribution of the particle group. From the calculation formula of α, it can be seen that the smaller α is, the smaller w is, and the larger $x_{c}$ is, which means that the particle size distribution tends to be narrow, and the most probable particle size tends to be large. Such a single aggregate grading and larger particle size will lead to the larger effective pore diameter, which is conducive to the improvement of the material’s water permeability; by contrast, the smaller α means that the particle size distribution tends to be wider and the most probable particle size tends to be smaller. Aggregates with such characteristics can play a good role in pore filling, with more continuous grading and fewer pores, and better strength. Although w=2σ, the coefficient of σ does not affect the trend in the horizontal comparison of α. Table 5 shows the parameters after the aggregate particle distribution fitting, the compressive strength data and water permeability rate data of permeable brick prepared with these 10 kinds of sand sample. As the mean value of flexural strength is 10.75 MPa, which is far greater than the 3.0 MPa specified in the national standard mentioned in Table 1, so it will not be discussed here.

Han et al. [43] prepared concrete with 4.75–9 mm coarse aggregate, and undertook image analysis after slicing. Although parameters such as aggregate size can also be obtained, the aggregate size distribution does not conform to the characteristics of normal distribution, which is due to the insufficient number of samples obtained by slicing after the preparation of concrete. The model proposed in this study can directly analyze the raw materials, and the aggregate particle size is small, the number of samples is extremely large and meets the statistical characteristics, so it is of guiding significance to obtain the characteristic parameters of fine-grained bulk materials.

#### 3.2.2. Aggregate Division Method

**Figure 13:**
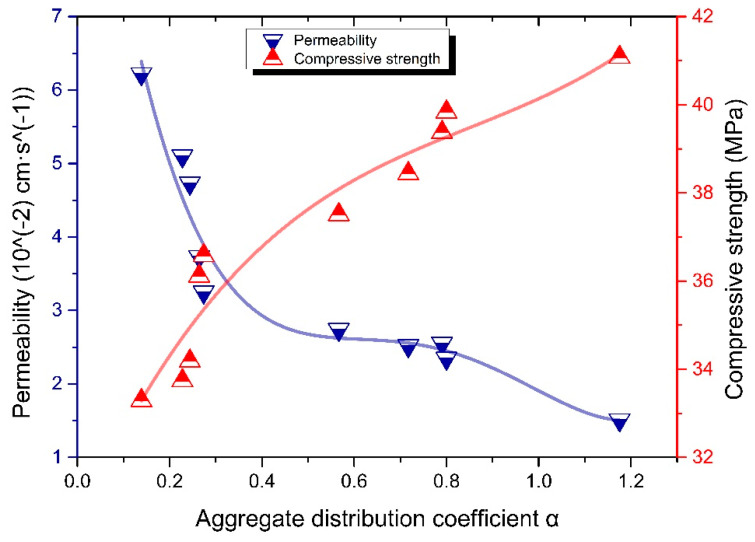
Relationship between α value of aggregate and compressive strength and water permeability of resin based permeable pavement materials.

The distribution width and the most probable particle size of each kind of aggregate are different, which makes the α value of 10 kinds of aggregate significantly different. As shown in Figure 13, the relationship between α of aggregate and compressive strength and water permeability rate of RBPB presents an X-shape; that is, with the increase of aggregate distribution coefficient α, the compressive strength increases and the water permeability rate decreases. Such X-shape is often seen in the study of porosity, that is, with the increase of porosity, the compressive strength and permeability rate of permeable materials show the opposite trend.

**Figure 14:**
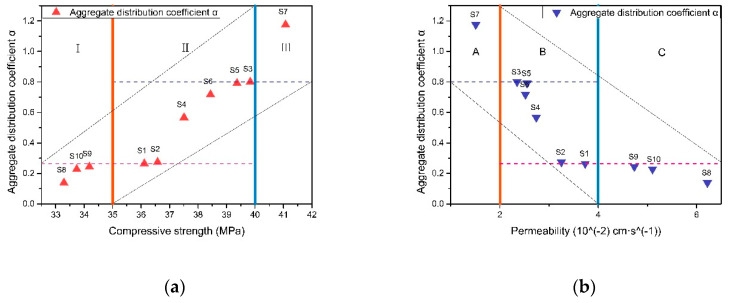
RBPB’s macro performance area division. (**a**). Relationship between compressive strength and aggregate distribution coefficient α. (**b**). Relationship between permeability and aggregate distribution coefficient α.

According to the compressive strength value and permeability rate, the characteristic parameter range of 10 kinds of sands is divided as shown in Figure 14a,b. Taking the strength boundary (35 MPa and 40 MPa) and permeability rate boundary (0.02 cm·s^−1^ and 0.04 cm·s^−1^) as the standard, the compressive strength of RBPB is divided into “I, Ⅱ, Ⅲ” three areas, and the permeability rate is divided into “A, B, C” three areas.

The characteristic parameter values of S1 and S3 are used as the classification nodes. The critical values in the two figures are compared and the intersection of them is taken. The aggregate distribution coefficient α value of aggregate is below 0.264, and the properties of RBPB prepared with it tend to be high permeable rate and low compressive strength (the compressive strength can still reach the national standard), which can be divided into high permeable aggregate (HPA); when the aggregate distribution coefficient α value of aggregate is above 0.8001, it is high compressive strength and low permeable rate (permeable rate can meet the national standard), which can be divided into high strength aggregate (HSA); when the aggregate distribution coefficient α is in the range of 0.264–0.8001, its strength and permeable rate are in a relatively balanced level, which can be divided into comprehensive aggregate (CA). S7 belongs to HSA, S8–S10 belongs to HPA and S1–S6 belongs to CA. The range of characteristic parameters of particle groups of various aggregates is shown in Table 6.

There are many studies to explore the mechanical properties and water permeability of porous materials from the porosity [26,44,45], but few studies from the perspective of particle group parameters. There are some defects in the design of permeable bricks only due to the parameters of the pores. Many properties of pores have influence on the strength and permeability of porous permeable material, such as pore size, pore distribution, pore shape, etc. Moreover, the key parameters of pore structure such as effective porosity, tortuosity and connectivity are difficult to obtain and the accuracy is low. This method of judging the performance of bricks by measuring porosity is a lagging method, that is, a material must be prepared to measure its performance, so a predictive model of predictability is required. This paper proposes a model to design the various functions of porous materials in advance by obtaining the particle group parameters of aggregates, which is beneficial to selecting materials and controlling materials in advance according to the design parameters.

### 3.3. Micromorphology Research Results of the Brick

#### 3.3.1. Apparent Morphology and Energy-Dispersive X-ray Spectroscopy (EDS)

**Figure 15:**
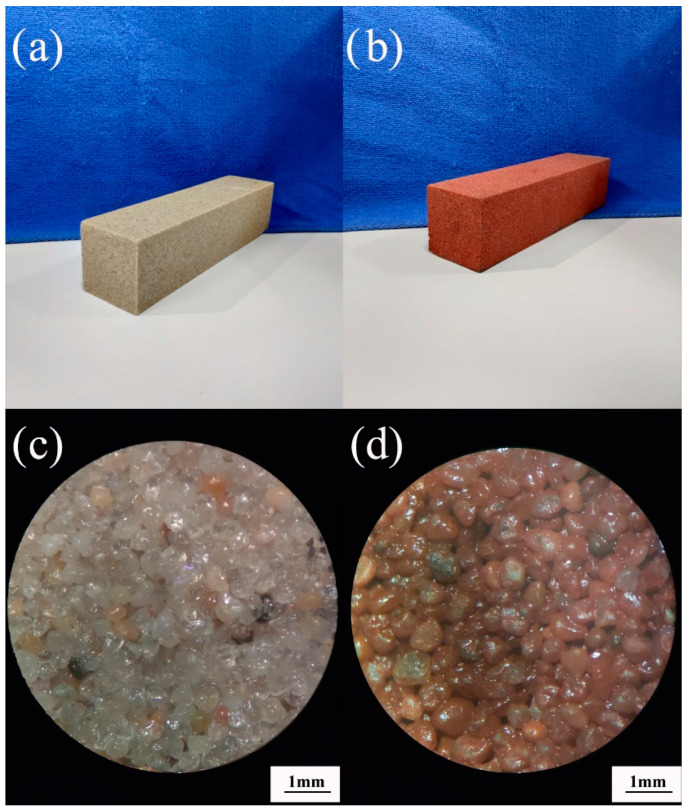
Photos of bricks before grinding: (**a**) permeable brick before dyeing; (**b**) permeable brick after dyeing; (**c**) micrograph of permeable brick before dyeing; (**d**) micrograph of permeable brick after dyeing.

According to the above research, we can know that S1–S6 is a comprehensive aggregate (CA), which has both good water permeability and good mechanical properties. Therefore, a unique representative aggregate, S4, located in the center of the comprehensive area is selected. The S4 aggregate and bisphenol-A epoxy resin is used to make bricks. S4 is the desert sand from Inner Mongolia. Figure 15a,b are the prepared brick strips, in which a small amount of red pigment is added to the sample shown in Figure 15b–d are the surface of the brick photographed with an optical microscope. From Figure 15, it can be seen that the roundness of the aggregate is very high, the aggregate flows and mixes easily in the brick making process, and the brick interior does not easily produce large pore defects that affect the strength. The brick surface is smooth, the pore distribution is uniform, and the pore diameter is small, which also makes the capillary phenomenon significant.

**Figure 16:**
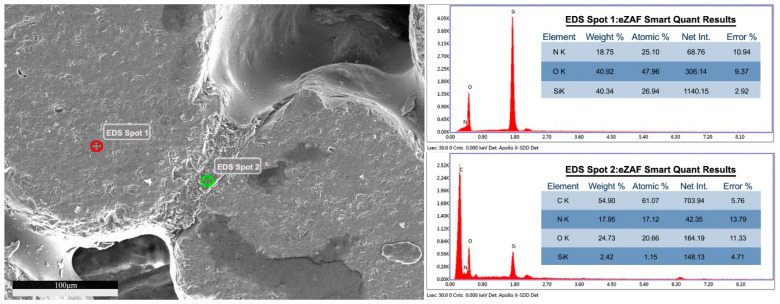
Energy-dispersive X-ray spectroscopy (EDS) analysis of the aggregate and the binder.

Energy-dispersive X-ray spectroscopy (EDS) was used to analyze the element composition and content of particles under SEM. It was found that the element composition of particles in the image is mainly silicon and oxygen, while the element composition of gap-filling material is mainly carbon, nitrogen and oxygen, which is in line with the experimental expectation, that is, the desert sand particle material is silicon dioxide, and the gap-filling binder material is cured epoxy resin, as shown in Figure 16.

#### 3.3.2. SEM Analysis

**Figure 17:**
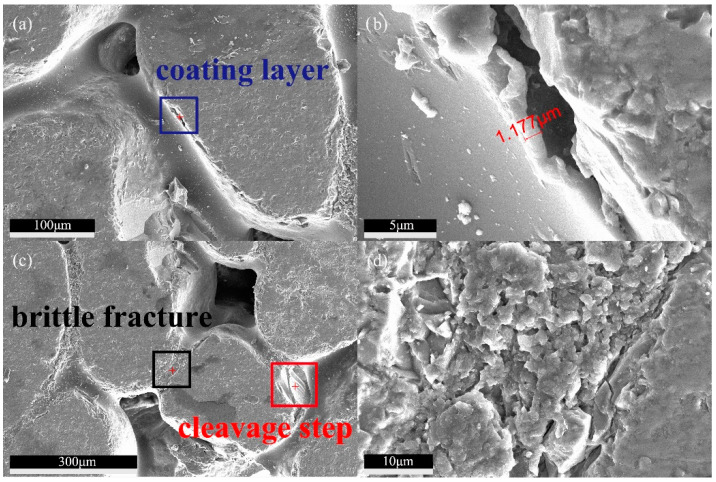
Characteristics of RBPB’s binder material. (**a**) Coating layer. (**b**) Thickness of coating layer. (**c**) Brittle fracture and cleavage step. (**d**) Brittle fracture details.

From Figure 17a, it can be seen that after resin liquid shrinks, it becomes the intermediate phase of point contact between particles, at the same time, it also provides a coating layer for particles, which is obviously visible in the blue square box. Figure 17b is an enlargement of the blue square box of Figure 17a, which shows that the thickness of the resin-coated layer is 1.177 μm. Figure 17c shows that the epoxy resin has obvious cleavage steps, in the red square box, after being damaged by external force, and the fracture surface is even and bright. Figure 17d is an enlargement of the black square box in Figure 17c, it can be seen that the epoxy resin produced a large number of brittle fracture defects during the process of cyclic shear stress (grinding). The above characteristics prove that this resin binder is hard and brittle, can withstand static pressure, and can meet the performance requirements of permeable bricks.

**Figure 18:**
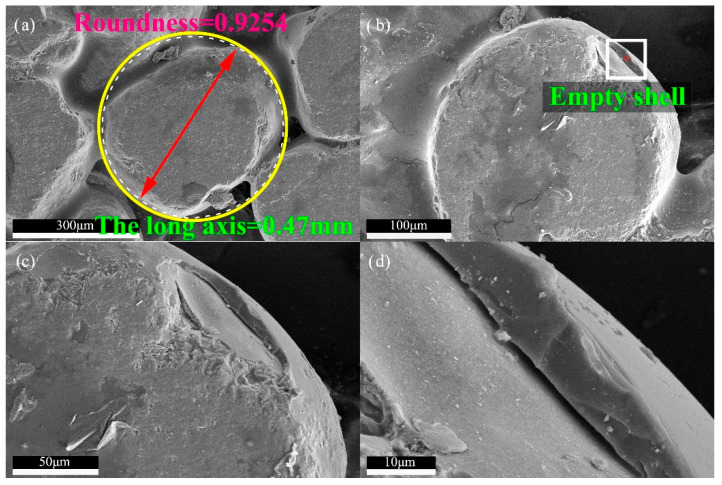
Apparent characteristics of RBPB’s aggregate and peelable surface defects. (**a**) Roundness and the length of the long axis of a particle. (**b**) Surface defect. (**c**) Empty shell. (**d**) Detail of the empty shell.

Image Pro Plus software was used to calculate more than 100,000 particles, the average roundness of this desert aeolian sand is 0.92. As shown in Figure 18, the roundness of one of the particles is about 0.93. From image analysis, it can be known that the long axis length of the particle is 0.47 mm, the short axis length is 0.34 mm, and the average value is 0.41 mm, in which the long axis length is close to value of $x_{c}$. The calculation formula of roundness is: the area of particle projection divided by the area of the circle with projection perimeter as its perimeter, the simplification formula is as in Equation (5):

(5) $$Roundness=\frac{4\times \pi \times Area}{Perimeter^{2}}$$

Figure 18a,b shows that the shape of aggregate is relatively round, and the roundness of desert sand particles is high, but there is still a shelling (empty shell) phenomenon on its surface due to weathering, as shown in Figure 18b–d. Its appearance will cause defects of brick and affect the strength of the brick. One study shows that surface treatment can effectively improve the interface bonding ability of sand [46], so surface treatment can be further used to eliminate the surface defects of sand.

**Figure 19:**
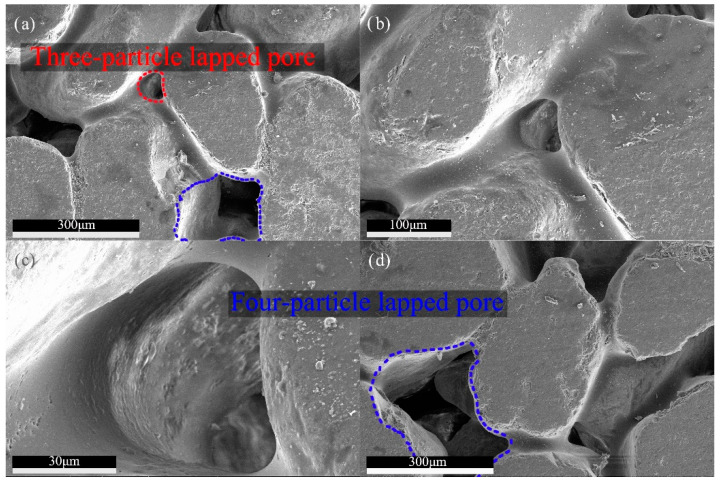
Pore structure characteristics of RBPB. (**a**) Three-particle overlapped pore. (**b**) Detail of the three-particle overlapped pore. (**c**) Internal detail of the three-particle overlapped pore. (**d**) Four-particle overlapped pore.

**Figure 20:**
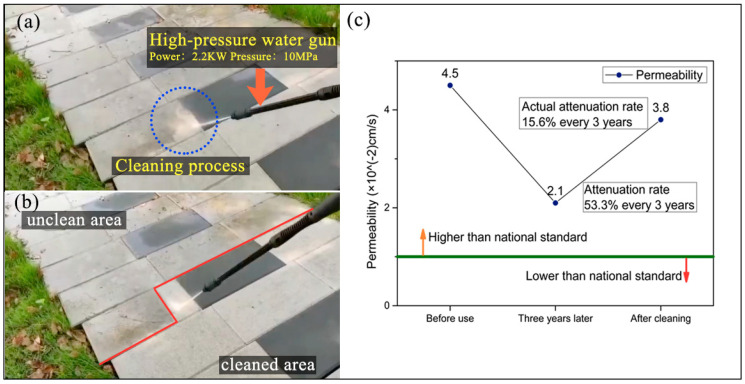
Long-term use of RBPB. (**a**) Cleaning process and tool of RBPB. (**b**) Comparison before and after cleaning. (**c**) Change of water permeability coefficient during use.

As shown in Figure 19, many smooth pores will be formed on the surface of the binder material due to the liquid shrinkage during the molding process, with a large number of pore sizes ranging from 30–100 μm. These pores can be divided into two types according to the contact form of the particles. One type is a three-sided pore formed by overlapping three particles, with a diameter of 30–50 microns, and the other type is formed by four particles overlapping, and the four-sided pores are about 100 microns wide. The three sides pore is affected by the resin to form a small round pore, while the four sides pore is affected by the overlapping state of the particles, as shown in Figure 19d, resulting in a large and square pore. Kayhanian’s [47] research on permeable concrete pavement shows that particles smaller than 38 μm in diameter are liable to cause clogging. Siriwardene [48] studied the migration of particles in porous materials, and found that the migration of particles with a diameter of 6 μm in pores leads to the formation of a clogging layer between permeable layer and soil, which is the real reason for the failure of permeable materials. The RBPB of the present invention has a micron-sized pore size, and the pore size is dozens of times smaller than that of a water-permeable material with a millimeter-level pore size. The two types of pore can more effectively prevent the migration of harmful particles in porous materials when blocking large particles. This kind of surface structure can effectively prolong the use period of permeable pavement. The surface-covered dust can be easily removed by a small high-pressure water gun. Figure 20a,b shows the cleaning of RBPB which has been used on the test road for 3 years. And the change of water permeability over time is shown in Figure 20c.

It can be seen from Figure 20a,b that the surface of RBPB is inevitably covered by silt and dust during use, but its good surface state can still be restored by simple washing with a small high-pressure water gun. In Figure 20c, the permeability of RBPB before use is $4.5\times 10^{-2}\;\mathrm{cm}/s$, but after three years of service, its permeability decays to $2.1\times 10^{-2}\;\mathrm{cm}/s$, and the attenuation rate is 53.3% every three years. After the surface was washed, its water permeability increased to $3.8\times 10^{-2}\;\mathrm{cm}/s$, and the attenuation rate was 15.6% every three years. If the calculation is based on an attenuation rate of 15.6% every three years, it will take 15 years for the permeability rate to decay below the national standard of $1.0\times 10^{-2}\;\mathrm{cm}/s$. In terms of durability, the wear resistance of RBPB can meet the requirements, and after 25 freeze-thaw cycles, the mass loss and strength loss are less than 13.5%.

Some studies have shown that [49,50] the proper pore size and the surface that can be wetted by the liquid are conducive to reducing the contact angle and increase the surface tension to form the capillary phenomenon, so that when the water contacts the brick surface, it can be wetted and expanded, and the water entering the hole is easy to move along the pores; the water permeability rate can be increased under the combined action of the pulling force generated by capillary action and the water’s own gravity, which is the reason why the water permeability rate of permeable brick made of fine aggregate can also exceed the national standard.

## 4. Conclusions

A kind of resin-based permeable brick with micron pore size was prepared by using the aggregate with a particle size range of 0.08–0.6 mm and bisphenol-A epoxy resin. The binder material and the characteristic parameters of aggregate particle group and pores of RBPB were studied. The results can be summarized in the following points:

(1) The tensile strength of bisphenol-A epoxy resin is 60.03 MPa, and the elongation at break is 6.17%. The glass transition temperature of bisphenol-A epoxy resin is 61 °C. The liquid shrinkage of the resin has a coating effect on the particles under SEM observation. The thickness of the coating layer is about 1 μm, and there is a brittle fracture characteristic when the resin is damaged by force. It is a hard and brittle adhesive material suitable for withstanding static pressure.
(2) An aggregate classification method was obtained: (a) when α < 0.264, its performance tends to be high permeable and low strength, which is divided into high permeable aggregate; (b) when α > 0.8001, its performance is high strength and low water permeability, which is divided into high-strength aggregate; (c) when the aggregate distribution coefficient α value is in the range of 0.264–0.8001, its strength performance and water permeability are in a relatively balanced level, which is a comprehensive aggregate; SEM analysis of the permeable brick aggregate prepared from desert sand shows that the roundness of the aggregate particles is very high, but due to weathering there will be surface defects of the empty shell, which may affect the strength of the RBPB; according to this study, permeable brick can be made from desert sand.
(3) Pores of RBPB can be divided into two types: three-sided pores and four-sided pores. The three side pore with a diameter of 30–50 microns, and the four side pores are about 100 microns wide. Permeable bricks with micron level pores have three advantages: (a) it can prevent large particles of sediment; (b) it can effectively reduce the migration of particles below 38 microns in the permeable brick, which is beneficial to prevent the formation of a clogging layer between the permeable brick and the base soil; and (c) the capillary phenomenon of small pores is obvious, facilitates the movement of water in the brick, and improves the macro-permeability.

## Figures and Tables

**Table 1:** Performance parameters of permeable bricks specified in national standard GB/T 25993-2010 [36].

Test Items	Flexural Strength (MPa)	Compressive Strength (MPa)	$\mathrm{Permeability}\;\mathrm{Rate}\;(\times 10^{-2}\;\mathrm{cm}/s)$
performance requirement	≥3.0	≥30.0	≥1.0

**Table 2:** The mix proportion of resin-based permeable brick (RBPB).

Composition	Sand	Resin	Curing Agent
Weight (g)	7617	352	105
Weight fraction (wt%)	94.3	4.4	1.3

**Table 3:** Tensile test mix proportion of binder materials.

Grade	Resin (g)	Curing Agent (g)	Total Weight (g)
E44	80	20	100

**Table 4:** Tensile test data.

Test Items	Maximum Force/N	Elongation at Break/%	Tensile Fracture Stress/MPa	Tensile Strength/MPa	Offset Yield Stress/MPa
Binder	3301.6	6.17	60.03	60.03	55.97

**Table 5:** Particle group distribution data and sample properties.

Sample	w	$x_{c}/\mathrm{mm}$	α	Compressive Strength/MPa	$\mathrm{Permeability}/\times 10^{-2}\;\mathrm{cm}/s$
**S1**	0.066	0.25	0.264	36.12	3.74
**S2**	0.0735	0.2686	0.2736	36.58	3.25
**S3**	0.1329	0.1661	0.8001	39.84	2.35
**S4**	0.2746	0.4844	0.5668	37.51	2.74
**S5**	0.2419	0.3059	0.7907	39.37	2.55
**S6**	0.131	0.1826	0.7174	38.44	2.52
**S7**	0.2098	0.1785	1.1753	41.08	1.51
**S8**	0.0697	0.5026	0.1386	33.29	6.22
**S9**	0.1042	0.4273	0.2438	34.19	4.73
**S10**	0.0823	0.3616	0.2276	33.74	5.1

**Table 6:** Performance index and particle group characteristic parameter range of three types of aggregates.

Aggregate Category	Permeability/cm·s^−1^	Compressive Strength/MPa	α
High permeable aggregate	≥0.040	≥30	<0.264
Comprehensive aggregate	≥0.020	≥35	0.264–0.8001
High strength aggregate	≥0.010	≥40	>0.8001

## References

[1] Cohen J.E. (2003). Human Population: The Next Half Century. Science.

[2] Meybeck M., Vörösmarty C. (2005). Fluvial Filtering of Land-To-Ocean Fluxes: From Natural Holocene Variations to Anthropocene. Cr. Geosci..

[3] Grimm N.B., Faeth S.H., Golubiewski N.E., Redman C.L., Wu J., Bai X., Briggs J.M. (2008). Global Change and the Ecology of Cities. Science.

[4] Walsh C.J., Fletcher T.D., Burns M.J. (2012). Urban Stormwater Runoff: A New Class of Environmental Flow Problem. PLoS ONE.

[5] Weng Q. (2001). Modeling Urban Growth Effects on Surface Runoff with the Integration of Remote Sensing and GIS. Environ. Manag..

[6] Jiang Y., Zevenbergen C., Ma Y. (2018). Urban Pluvial Flooding and Stormwater Management: A Contemporary Review of China’s Challenges and "Sponge Cities" Strategy. Environ. Sci. Policy.

[7] Jamali B., Löwe R., Bach P.M., Urich C., Arnbjerg-Nielsen K., Deletic A. (2018). A Rapid Urban Flood Inundation and Damage Assessment Model. J. Hydrol..

[8] Driscoll M., Clinton S., Jefferson A., Manda A., McMillan S. (2010). Urbanization Effects on Watershed Hydrology and In-Stream Processes in the Southern United States. Water Sui..

[9] Cao C., Lee X., Liu S., Schultz N., Xiao W., Zhang M., Zhao L. (2016). Urban Heat Islands in China Enhanced by Haze Pollution. Nat. Commun..

[10] Connors J.P., Galletti C.S., Chow W.T.L. (2013). Landscape Configuration and Urban Heat Island Effects: Assessing the Relationship between Landscape Characteristics and Land Surface Temperature in Phoenix, Arizona. Landscape Ecol..

[11] Sen S., Roesler J. (2019). Thermal and Optical Characterization of Asphalt Field Cores for Microscale Urban Heat Island Analysis. Constr. Build. Mater..

[12] Li H., Harvey J., Ge Z. (2014). Experimental Investigation On Evaporation Rate for Enhancing Evaporative Cooling Effect of Permeable Pavement Materials. Constr. Build. Mater..

[13] Strecker E.W., Quigley M.M., Urbonas B.R., Jones J.E., Clary J.K. (2001). Determining Urban Storm Water BMP Effectiveness. J. Water Res. Plan. Man..

[14] Dietz M.E. (2007). Low Impact Development Practices: A Review of Current Research and Recommendations for Future Directions. Water Air Soil Pollut..

[15] Norton B.A., Coutts A.M., Livesley S.J., Harris R.J., Hunter A.M., Williams N.S.G. (2015). Planning for Cooler Cities: A Framework to Prioritise Green Infrastructure to Mitigate High Temperatures in Urban Landscapes. Landscape Urban Plan..

[16] Abbott C.L., Comino Mateos L. (2003). In-Situ Hydraulic Performance of a Permeable Pavement Sustainable Urban Drainage System. Water Environ. J..

[17] Coutts A.M., Tapper N.J., Beringer J., Loughnan M., Demuzere M. (2013). Watering Our Cities: Thecapacity for Water Sensitive Urban Design to Support Urban Cooling and Improve Human Thermal Comfort in the Australian Context. Prog. Phys. Geogr. Earth Environ..

[18] Van Roon M. (2007). Water Localisation and Reclamation: Steps towards Low Impact Urban Design and Development. J. Environ. Manag..

[19] Lim H.S., Lu X.X. (2016). Sustainable Urban Stormwater Management in the Tropics: An Evaluation of Singapore’S ABC Waters Program. J. Hydrol..

[20] Xia J., Zhang Y., Xiong L., He S., Wang L., Yu Z. (2017). Opportunities and Challenges of the Sponge City Construction Related to Urban Water Issues in China. Sci. China Earth Sci..

[21] Fletcher T.D., Shuster W., Hunt W.F., Ashley R., Butler D., Arthur S., Trowsdale S., Barraud S., Semadeni-Davies A., Bertrand-Krajewski J. (2015). SUDS, LID, BMPs, WSUD and More—the Evolution and Application of Terminology Surrounding Urban Drainage. Urban Water J..

[22] Scholz M., Grabowiecki P. (2007). Review of Permeable Pavement Systems. Build. Environ..

[23] Jiang W., Sha A., Xiao J., Li Y., Huang Y. (2015). Experimental Study On Filtration Effect and Mechanism of Pavement Runoff in Permeable Asphalt Pavement. Constr. Build. Mater..

[24] Wang Y., Gao S., Liu X., Tang B., Mukiza E., Zhang N. (2019). Preparation of Non-Sintered Permeable Bricks Using Electrolytic Manganese Residue: Environmental and NH3-N Recovery Benefits. J. Hazard. Mater..

[25] Lin C., Wu C., Ho H. (2006). Recovery of Municipal Waste Incineration Bottom Ash and Water Treatment Sludge to Water Permeable Pavement Materials. Waste Manag..

[26] Lian C., Zhuge Y., Beecham S. (2011). The Relationship between Porosity and Strength for Porous Concrete. Constr. Build. Mater..

[27] Tang C., Cheng C., Tsai C. (2019). Mix Design and Mechanical Properties of High-Performance Pervious Concrete. Materials.

[28] Elizondo-Martinez E.J., Andres-Valeri V.C., Rodriguez-Hernandez J., Castro-Fresno D. (2019). Proposal of a New Porous Concrete Dosage Methodology for Pavements. Materials.

[29] Sansalone J., Kuang X., Ying G., Ranieri V. (2012). Filtration and Clogging of Permeable Pavement Loaded by Urban Drainage. Water Res..

[30] Tang B., Gao S., Wang Y., Liu X., Zhang N. (2019). Pore Structure Analysis of Electrolytic Manganese Residue Based Permeable Brick by Using Industrial CT. Constr. Build. Mater..

[31] Zhu M., Wang H., Liu L., Ji R., Wang X. (2017). Preparation and Characterization of Permeable Bricks from Gangue and Tailings. Constr. Build. Mater..

[32] Zhou C. (2018). Production of Eco-Friendly Permeable Brick from Debris. Constr. Build. Mater..

[33] Yuan X., Tang Y., Li Y., Wang Q., Zuo J., Song Z. (2018). Environmental and Economic Impacts Assessment of Concrete Pavement Brick and Permeable Brick Production Process—A Case Study in China. J. Clean. Prod..

[34] Mora C.F., Kwan A.K.H. (2000). Sphericity, Shape Factor, and Convexity Measurement of Coarse Aggregatefor Concrete Using Digital Image Processing. Cem. Concr. Res..

[35] Kwan A.K.H., Mora C.F., Chan H.C. (1999). Particle Shape Analysis of Coarse Aggregate Using Digital Image Processing. Cem. Concr. Res..

[36] (2010). Permeable Paving Bricks & Permeable Paving Flags.

[37] (2011). Sand for Construction.

[38] (2011). State Administration for Quality Supervision and Inspection and Quarantine Permeable Paving Bricks and Permeable Paving Flags.

[39] (2012). Sand-Based Water Permeable Brick.

[40] (2008). Test Method for Properties of Resin Casting Body.

[41] Van Mier J.G.M., Meyer D., Man H. (2008). Fracture of Quasi-Brittle Materials Like Concrete under Compressive Loading. Adv. Mat. Res..

[42] Zhou J., Lucas J.P. (1999). Hygrothermal Effects of Epoxy Resin. Part II: Variations of Glass Transition Temperature. Polymer.

[43] Han J., Wang K., Wang X., Monteiro P.J.M. (2016). 2D Image Analysis Method for Evaluating Coarse Aggregate Characteristic and Distribution in Concrete. Constr. Build. Mater..

[44] Chindaprasirt P., Hatanaka S., Chareerat T., Mishima N., Yuasa Y. (2008). Cement Paste Characteristics and Porous Concrete Properties. Constr. Build. Mater..

[45] Ba M., Qian C., Guo X., Han X. (2011). Effects of Steam Curing On Strength and Porous Structure of Concrete with Low Water/Binder Ratio. Constr. Build. Mater..

[46] Rodriguez E.L., Newaz G.M. (1988). The Effect of Sand Surface Treatment on Sand-Filled Composites. Polym. Compos..

[47] Kayhanian M., Anderson D., Harvey J.T., Jones D., Muhunthan B. (2012). Permeability Measurement and Scan Imaging to Assess Clogging of Pervious Concrete Pavements in Parking Lots. J. Environ. Manag..

[48] Siriwardene N., Deletic A., Fletcher T. (2007). Clogging of Stormwater Gravel Infiltration Systems and Filters: Insights from a Laboratory Study. Water Res..

[49] Wardlaw N.C., Li Y., Forbes D. (1987). Pore-Throat Size Correlation from Capillary Pressure Curves. Transp. Porous Med..

[50] Karkare M.V., Fort T. (1993). Water Movement in "Unsaturated" Porous Media Due to Pore Size and Surface Tension Induced Capillary Pressure Gradients. Langmuir.

